# Contraceptive use and unplanned pregnancy among female sex workers in Zambia^☆^^☆☆^^★^

**DOI:** 10.1016/j.contraception.2017.07.003

**Published:** 2017-09

**Authors:** Michael M. Chanda, Katrina F. Ortblad, Magdalene Mwale, Steven Chongo, Catherine Kanchele, Nyambe Kamungoma, Leah G. Barresi, Guy Harling, Till Bärnighausen, Catherine E. Oldenburg

**Affiliations:** a John Snow, Inc, Lusaka, Zambia; b Department of Global Health and Population, Harvard T.H. Chan School of Public Health, Boston, MA, USA; c Department of Epidemiology, Harvard T.H. Chan School of Public Health, Boston, MA, USA; d Research Department of Infection and Population Health, University College London, UK; e Africa Health Research Institute, Mtubatuba, South Africa; f Institute of Public Health, Faculty of Medicine, University of Heidelberg, Heidelberg, Germany; g Francis I. Proctor Foundation, University of California, San Francisco, CA, USA

**Keywords:** Female sex workers, Abortion, Family planning, Sub-Saharan Africa

## Abstract

**Objectives:**

Access to reproductive healthcare, including contraceptive services, is an essential component of comprehensive healthcare for female sex workers (FSW). Here, we evaluated the prevalence of and factors associated with contraceptive use, unplanned pregnancy, and pregnancy termination among FSW in three transit towns in Zambia.

**Study design:**

Data arose from the baseline quantitative survey from a randomized controlled trial of HIV self-testing among FSW. Eligible participants were 18 years of age or older, exchanged sex for money or goods at least once in the past month, and were HIV-uninfected or status unknown without recent HIV testing (<3 months). Logistic regression models were used to assess factors associated with contraceptive use and unplanned pregnancy.

**Results:**

Of 946 women eligible for this analysis, 84.1% had been pregnant at least once, and among those 61.6% had an unplanned pregnancy, and 47.7% had a terminated pregnancy. Incarceration was associated with decreased odds of dual contraception use (aOR=0.46, 95% CI 0.32–0.67) and increased odds of unplanned pregnancy (aOR=1.75, 95% CI 1.56–1.97). Condom availability at work was associated with increased odds of using condoms only for contraception (aOR=1.74, 95% CI 1.21–2.51) and decreased odds of unplanned pregnancy (aOR=0.63, 95% CI 0.61–0.64).

**Conclusions:**

FSW in this setting have large unmet reproductive health needs. Structural interventions, such as increasing condom availability in workplaces, may be useful for reducing the burden of unplanned pregnancy.

## 1 Introduction

Female sex workers (FSW) in sub-Saharan Africa face large barriers to accessing essential healthcare services, including stigma and discrimination, criminalization of sex work and fear of repercussions of seeking care, and challenges with logistically accessing care (e.g., difficulty with transportation or clinic hours). FSW bear a disproportionately large burden of HIV in sub-Saharan Africa [1]. Factors at multiple levels, including structural environments and interpersonal dynamics, potentiate this elevated risk among FSW [2]. An unmet need for contraception has been documented in several settings in sub-Saharan Africa among FSW [3], [4], [5], [6]. Lack of access to contraceptive services may lead to unplanned pregnancies or pregnancy termination [4], which can in turn lead to increased maternal mortality, continued dependence on sex work to financially support children, or HIV transmission to children if prevention of mother-to-child transmission services are not utilized.

Unsafe abortion is a major cause of maternal mortality worldwide [7]. Despite provisions for the legality of abortion under some circumstances in Zambia, such as in the case of risk to the pregnant woman's life, physical, or mental health, in the case of child rape, or in the case of high risk of fetal impairment, abortion remains a major contributor to maternal mortality [8]. Increasing access to contraception may reduce unplanned pregnancy and pregnancy termination, which could in turn lead to reduced maternal mortality and improved birth outcomes.

In Zambia, little evidence exists of contraception use and pregnancy outcomes among FSW. A better understanding of contraceptive access and burden of unplanned pregnancy and pregnancy termination may lead to improved contraceptive services for this population. Here, we report the prevalence of and factors associated with contraceptive access and unplanned and terminated pregnancy among a sample of FSW not known to be living with HIV in three transit towns in Zambia.

## 2 Methods

### 2.1 Participants and procedures

We used data from the baseline cross-sectional quantitative assessment of the Zambian Peer Educators for HIV Self-Testing (ZEST) Study, a cluster randomized trial investigating the effect of HIV self-testing provision among HIV testing outcomes among female sex workers in Zambian transit towns (ClinicalTrials.gov NCT02827240) [9]. Participants completed the baseline questionnaire from September to October, 2016. Participants were recruited by peer educators, who were current or former female sex workers in Livingstone, Chirundu, and Kapiri Mposhi, Zambia. Peer educators were recruited through contacts with former sex worker organizations working in each of the three study towns. Each peer educator recruited approximately six women who were enrolled in the trial.

Women were eligible for participation in the study if they were 18 years of age or over at the time of enrollment, reported exchanging sex for money, goods, or other items of value at least once in the prior month, self-reported an HIV negative or unknown status and had not tested for HIV in the past 3 months, and reported a primary residence in one of the three study sites (Chirundu, Livingstone, or Kapiri Mposhi). The study was reviewed and approved by the Institutional Review Boards at the Harvard T.H. Chan School of Public Health in Boston, MA, USA, and ERES Converge in Lusaka, Zambia. We obtained written informed consent from all participants.

### 2.2 Measures

A trained research assistant collected all data in a face-to-face interview at a location that was convenient and private. Data were collected via tablet using the electronic data capture platform CommCare (Dimagi, Inc. Cambridge, MA, USA).

*Contraceptive use* was measured by asking participants if they were currently using a method for family planning to avoid becoming pregnant. We asked those who answered affirmatively what method they were using as an open-ended question. Research assistants checked all that applied from a pre-populated list, or wrote in other categories in an open text field. We asked those who reported they were not currently using family planning what were some reasons they were not using family planning in an open-ended fashion. Research assistants recorded outcomes on a pre-populated list or wrote in other categories in an open text field. We considered participants to be using only condoms for contraception if they reported condom use for contraception but no other contraceptive method. We considered participants to be using non-barrier methods of contraception only if they reported current use of oral birth control pills, injectable contraceptives, implant devices, intrauterine devices or vaginal rings and did not report using condoms for contraception. We considered participants to be using dual contraceptive methods if they reported using both a non-barrier method and condoms for birth control purposes.

*Reproductive health outcomes*, including unplanned pregnancy and pregnancy termination, were measured by first asking participants if they had ever been pregnant. We asked those who responded affirmatively how many times they had been pregnant and how many living children they had. We asked women who reported a history of pregnancy if they had ever been pregnant when they did not want to be. We measured abortion by asking women “Have you ever been in a position where you or someone else has had to do something to end your pregnancy”.

*Demographics* included age, having a primary partner, if the participant could read and write, if they had a mobile phone, and monthly income in kwacha.

*Structural and Interpersonal* measures included arrest/incarceration history (measured by asking participants if they had ever been arrested or incarcerated), age at sexual debut, experiences of sexual assault by clients and partners in the past 12 months, and condom availability while working.

### 2.3 Statistical analysis

Individuals who reported that they were not using contraception because they were trying to become pregnant were excluded from all analyses. Distributions of characteristics were calculated with medians and interquartile ranges (IQR) for continuous variables and proportions for categorical variables. We investigated factors associated with (1) use of condoms only for contraception, (2) use of non-barrier methods only for contraception, (3) use of both condoms and non-barrier methods for contraception, (4) history of unplanned pregnancy, and (5) history of terminated pregnancy using logistic regression models, and included sociodemographic variables (including age, having a primary partner, literacy, mobile phone ownership, monthly income) and structural-level variables (including age at sexual debut, arrest/incarceration history, sexual abuse from clients and primary partners, and condom availability at workplaces). We included covariates that were theoretically associated with reproductive health outcomes that represented opportunities for intervention development (e.g., identifying subgroups of participants with whom interventions could be focused or identifying potentially modifiable risk factors). Models of unplanned or terminated pregnancy were restricted only to women who reported ever being pregnant. Standard errors were adjusted for clustering within study site. All analyses were conducted in Stata 14.1 (StataCorp, College Station, TX, USA).

## 3 Results

A total of 1280 women underwent phone screening, and 965 (75.4%) were eligible and consented to participate in the study. Reasons for exclusion were self-reported HIV infection (*N*=174; 13.6%), HIV testing in the past 3 months (*N*=163; 12.7%), not meeting the sex work definition (*N*=157; 12.3%), not willing to participate (*N*=104; 10.8%), under age 18 (*N*=85; 6.6%), and residing outside of the study catchment area (*N*=84; 6.6%). One woman could have multiple reasons for exclusion. Of the 965 women who enrolled in the study and participated in the baseline survey, 19 (2.0%) reported that they were trying to get pregnant and were excluded from this analytic sample.

**Fig. 1:**
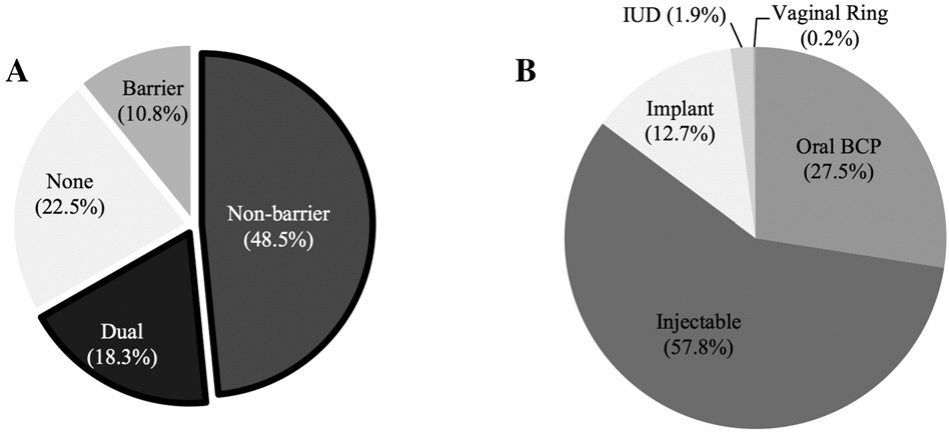
Proportion of female sex workers reporting contraceptive use (1A) and types of non-barrier methods reported (1B) among individuals using non-barrier contraception (*N*=946). Abbreviations: IUD, intrauterine device; BCP; birth control pill.

Table 1 displays descriptive characteristics for the 946 women in the analytic sample. Median age was 25 years (IQR 21–30). Half (50.0%) were recruited in Livingstone, and approximately one-quarter each were recruited in Chirundu and Kapiri Mposhi. More than half (59.4%) reported having a primary partner and most (84.1%) had at least one lifetime pregnancy. Approximately two-thirds (66.6%) were currently using a form of non-barrier birth control (Fig. 1A). The most common non-barrier methods included injectables (57.8%), oral birth control pills (27.5%), and implants (12.7%; Fig. 1B). More than half (61.7%) of participants who had been pregnant at least once had had an unplanned pregnancy, and 47.7% reported terminating a pregnancy.

**Table 1:** Descriptive characteristics of women using and not using a non-barrier family planning method among female sex workers in Zambia

	Using NB Method (*N*=630)	Not Using NB Method (*N*=316)	Overall (*N*=946)	p Value^1^
Age (median, IQR)	24 (21–29)	26 (21–32)	25 (21–30)	.005
Site				
Livingstone	318 (67.2%)	155 (32.8%)	473 (50.0%)	.71
Chirundu	162 (67.5%)	78 (32.5%)	240 (25.4%)	
Kapiri	150 (64.4%)	83 (35.6%)	233 (24.6%)	
Have a primary partner	379 (67.6%)	182 (65.4%)	561 (59.4%)	.48
Can read and write	489 (68.9%)	221 (60.0%)	710 (75.5%)	.02
Mobile phone ownership	545 (67.7%)	260 (60.3%)	805 (85.1%)	.10
Monthly income				
No income	121 (61.7%)	75 (38.3%)	196 (21.1%)	.25
<250 kwacha^2^	79 (67.5%)	38 (32.5%)	117 (12.6%)	
251–500 kwacha^2^	149 (64.0%)	84 (36.1%)	233 (25.1%)	
501–1000 kwacha^2^	174 (71.9%)	68 (28.1%)	242 (26.0%)	
1001–1500 kwacha^2^	51 (70.8%)	21 (29.2%)	72 (7.7%)	
>1500 kwacha^2^	48 (68.6%)	75 (38.3%)	70 (7.5%)	
Number of living children	1 (1–2)	1 (1–2)	1 (1–2)	.008
Age at sexual debut (median, IQR)	16 (15–18)	16 (15–18)	16 (15–18)	.70
Ever arrested/incarcerated	176 (64.9%)	95 (67.4%)	271 (28.7%)	.49
Client sexual abuse, past 12 months	178 (67.2%)	87 (66.5%)	265 (28.0%)	.88
Primary partner sexual abuse, past 12 months	126 (68.1%)	59 (66.3%)	185 (19.6%)	.67
Condom availability at work				
Never	11 (64.7%)	6 (35.3%)	17 (1.8%)	.88
Seldom	30 (71.4%)	12 (28.6%)	42 (4.5%)	
Sometimes	388 (65.7%)	203 (34.4%)	591 (62.6%)	
Often	55 (70.5%)	23 (29.5%)	78 (8.3%)	
Always	145 67.1%)	71 (32.9%)	216 (22.9%)	
Ever been pregnant	553 (69.5%)	243 (30.5%)	796 (84.1%)	<.001
Number of pregnancies (median, IQR)^3^	2 (1–3)	2 (1–3)	2 (1–3)	.10
Number of living children (median, IQR)^3^	1 (1–2)	1 (1–2)	1 (1–2)	>.99
Any unplanned pregnancy^3^	351 (71.6%)	139 (28.4%)	490 (61.6%)	.10
Any terminated pregnancy^3^	276 (72.6%)	104 (27.4%)	380 (47.7%)	.07

Abbreviations: NB, non-barrier; IQR, interquartile range ^1^Wilcoxon rank-sum test for continuous variables or Fisher's exact test for categorical variables; ^2^10 kwacha = approximately USD$1; ^3^Among women who had ever been pregnant (*N*=796).

Table 2 lists results of models assessing factors associated with contraceptive use. Older age at sexual debut was associated with increased odds of using only condoms as contraception (aOR 1.04, 95% CI 1.02–1.06), but was not associated with using only non-barrier methods or use of both condoms and non-barrier methods. Condom availability at work was associated with increased odds of using only condoms for contraception (aOR 1.74, 95% CI 1.21–2.51). History of incarceration or arrest was associated with decreased odds of using both condom and non-barrier methods for contraception (aOR 0.46, 95% CI 0.32–0.67).

**Table 2:** Association between history of unplanned and terminated pregnancy and current contraceptive use among female sex workers in Zambia with no current fertility desire (*N*=946).

	Condoms only		Non-barrier only		Condoms and non-barrier methods	
	*Bivariate*^*1*^ OR (95% CI)	*Multivariable*^*1*^ aOR (95% CI)	*Bivariate*^*1*^ OR (95% CI)	*Multivariable*^*1*^ aOR (95% CI)	*Bivariate*^*1*^ OR (95% CI)	*Multivariable*^*1*^ aOR (95% CI)
Age	1.02 (0.97–1.07)	1.04 (0.99–1.09)	0.96 (0.95–0.97)	0.93 (0.91–0.95)	1.01 (0.97–1.05)	0.98 (0.93–1.03)
Have a primary partner	0.98 (0.58–1.66)	1.09 (0.57–2.07)	1.10 (0.97–1.25)	1.01 (0.84–1.22)	1.50 (0.66–3.37)	1.73 (0.91–3.30)
Can read and write	0.90 (0.52–1.55)	0.96 (0.83–1.11)	1.48 (1.02–2.14)	1.25 (0.80–1.94)	1.73 (0.75–4.00)	1.86 (0.60–5.76)
Mobile phone ownership	1.12 (0.41–3.08)	1.02 (0.40–2.59)	1.38 (1.23–1.56)	1.45 (1.33–1.59)	1.63 (1.50–1.77)	1.64 (1.15–2.33)
Monthly income						
No income	1.00	1.00	1.00	1.00	1.00	1.00
<250 kwacha	1.19 (0.61–2.32)	1.17 (0.67–2.05)	1.29 (0.92–1.80)	1.16 (0.86–1.55)	14.6 (6.50–32.6)	15.2 (5.62–40.9)
251–500 kwacha	0.35 (0.15–0.81)	0.37 (0.18–0.75)	1.10 (0.58–2.09)	1.04 (0.57–1.90)	4.12 (1.60–11.0)	3.61 (1.23–10.6)
501–1000 kwacha	0.27 (0.16–0.46)	0.24 (0.16–0.35)	1.59 (0.97–2.59)	1.48 (0.77–2.83)	2.95 (0.89–9.79)	2.68 (0.47–15.2)
1001–1500 kwacha	0.55 (0.22–1.42)	0.48 (0.23–0.43)	1.51 (0.95–2.38)	1.35 (0.72–2.54)	2.19 (0.59–8.09)	1.74 (0.28–10.9)
>1500 kwacha	0.39 (0.27–0.57)	0.31 (0.22–0.43)	1.35 (0.62–2.97)	1.16 (0.44–3.10)	1.18 (0.43–3.27)	1.05 (0.21–5.37)
Number of living children	0.95 (0.84–1.07)	0.84 (0.78–0.91)	1.11 (0.99–1.23)	1.39 (1.13–1.71)	1.25 (0.92–1.70)	1.40 (1.04–1.87)
Age at sexual debut	1.08 (1.06–1.11)	1.04 (1.02–1.06)	0.98 (0.92–1.03)	0.98 (0.95–1.01)	1.04 (1.00–1.08)	1.01 (0.93–1.10)
Ever arrested/incarcerated	0.79 (0.51–1.22)	0.79 (0.49–1.26)	0.90 (0.75–1.07)	0.95 (0.85–1.07)	0.46 (0.21–1.01)	0.46 (0.32–0.67)
Client sexual abuse, past 12 months	0.75 (0.46–1.23)	0.74 (0.32–1.74)	1.03 (0.73–1.46)	1.09 (0.84–1.43)	1.83 (0.75–4.49)	2.20 (0.92–5.29)
Primary partner sexual abuse, past 12 months	1.03 (0.42–2.51)	1.59 (0.74–3.41)	1.08 (0.88–1.33)	0.98 (0.70–1.38)	1.30 (0.34–4.93)	1.22 (0.27–5.57)
Condoms always available at work	1.56 (1.00–2.42)	1.74 (1.21–2.51)	1.03 (0.75–1.41)	1.07 (0.79–1.45)	0.98 (0.77–1.23)	1.25 (0.71–2.23)

OR, odds ratio; aOR, adjusted odds ratio; 95% CI, 95% confidence interval. ^1^Logistic regression model with standard errors accounting for clustering within study site.

Table 3 lists results of models assessing factors associated with history of unplanned and terminated pregnancy. Earlier age at sexual debut was associated with reduced odds of unplanned (aOR 0.91 per one-year increase in age at sexual debut, 95% CI 0.88–0.93) and terminated (aOR 0.94 per one-year increase in age at sexual debut, 95% CI 0.91–0.96) pregnancy. Condom availability in the workplace was associated with decreased odds of unplanned pregnancy (aOR 0.63, 95% CI 0.61–0.64) and termination of pregnancy (aOR 0.68, 95% CI 0.51–0.91). Arrest/incarceration history was associated with greater odds of unplanned pregnancy (aOR 1.75, 95% CI 1.56–1.97) and termination of pregnancy (aOR 1.87, 95% CI 1.64–2.12).

**Table 3:** Factors associated with a) unplanned pregnancy and b) pregnancy termination among female sex workers in Zambia who reported being pregnant at least once with no current fertility desire (*N*=796).

	Unplanned Pregnancy		Pregnancy Termination	
	*Bivariate*^*1*^ OR (95% CI)	*Multivariable*^*1*^ aOR (95% CI)	*Bivariate*^*1*^ OR (95% CI)	*Multivariable*^*1*^ aOR (95% CI)
Age	0.97 (0.94–0.99)	0.94 (0.93–0.96)	0.98 (0.96–1.01)	0.98 (0.97–0.98)
Have a primary partner	1.37 (0.77–2.47)	1.09 (0.67–1.80)	1.65 (0.99–2.74)	1.46 (0.94–2.23)
Can read and write	1.08 (0.69–1.67)	1.04 (0.59–1.85)	1.37 (1.08–1.74)	1.57 (1.32–1.85)
Mobile phone ownership	1.34 (1.02–1.76)	1.78 (1.57–2.02)	0.99 (0.65–1.52)	1.14 (0.82–1.58)
Monthly income				
No income	1.00	1.00	1.00	1.00
<250 kwacha	0.58 (0.19–1.76)	0.64 (0.20–2.10)	0.59 (0.16–2.24)	0.64 (0.18–2.26)
251–500 kwacha	1.44 (0.52–3.97)	1.32 (0.49–3.56)	1.69 (0.56–5.12)	1.42 (0.50–4.05)
501–1000 kwacha	2.09 (1.28–3.41)	2.24 (1.17–4.31)	1.92 (1.25–2.97)	1.81 (1.29–2.55)
1001–1500 kwacha	2.47 (1.94–3.15)	3.01 (1.45–6.23)	2.21 (1.06–4.60)	2.49 (1.73–3.60)
>1500 kwacha	1.57 (1.12–2.20)	1.88 (0.96–3.68)	1.02 (0.37–2.85)	1.06 (0.46–2.43)
Number of living children	1.06 (0.90–1.24)	1.30 (1.16–1.45)	1.07 (0.86–1.34)	1.18 (0.96–1.45)
Age at sexual debut	0.88 (0.86–0.90)	0.91 (0.88–0.93)	0.92 (0.89–0.94)	0.94 (0.91–0.96)
Ever arrested/incarcerated	1.75 (1.54–1.99)	1.75 (1.56–1.97)	1.96 (1.48–2.59)	1.87 (1.64–2.12)
Client sexual abuse, past 12 months	1.54 (0.90–2.64)	1.47 (0.72–3.00)	1.75 (0.71–4.29)	1.69 (0.62–4.60)
Primary partner sexual abuse, past 12 months	1.76 (1.18–2.63)	1.44 (0.98–2.12)	2.15 (1.29–3.57)	1.73 (0.94–3.20)
Condoms always available at work	0.61 (0.42–0.88)	0.63 (0.61–0.64)	0.63 (0.42–0.95)	0.68 (0.51–0.91)

OR, odds ratio; aOR, adjusted odds ratio; 95% CI, 95% confidence interval. ^1^Logistic regression model with standard errors accounting for clustering within study site.

## 4 Discussion

In this study of FSW in transit towns in Zambia with historically high HIV prevalence, we found a high prevalence of unplanned and terminated pregnancy. However, nearly three-quarters of women reported using a method of contraception, which is considerably higher than women in the general population in Zambia (approximately 25%) [10]. Approximately two-thirds of participants reported using a non-barrier method for contraception, but fewer than 20% of participants indicated they were using both condoms and a non-barrier method. For women without current pregnancy intention, access to and use of dual protection (the simultaneous use of both condoms and female-controlled modern non-barrier method) is an essential component of comprehensive reproductive health services [11], [12]. Modern non-barrier methods are the most effective for prevention of pregnancy, but do not reduce the risk of HIV acquisition. Condom use reduces the risk of HIV acquisition and thus is complementary to non-barrier methods. In the present study, it is possible that women who reported using both condoms and non-barrier methods were not using both simultaneously, and the true prevalence of dual contraceptive use may be even lower. These results indicate that interventions to improve access and use of condoms and non-barrier methods may help improve reproductive health outcomes among FSW in Zambian transit regions and other similar regions of sub-Saharan Africa.

The availability of condoms in the work place was associated with increased use of condoms as a family planning method and decreased unplanned pregnancy and termination of pregnancy. This may be because women working in venues where condoms are available may have greater agency with condom negotiation or increased access to reproductive health services. Furthermore, condom availability is likely a facilitator for condom use with clients, which may increase use and thus reduce unplanned pregnancies and thus termination of pregnancy. However, overall reported condom use was low in this population, which likely increases risk of acquisition of HIV and potentially mother-to-child transmission. Increasing availability of condoms in the work place may be a low-cost intervention to improve condom use and improve reproductive health outcomes for FSW.

Arrest/incarceration history was also an important determinant of contraceptive use and unplanned and terminated pregnancy. Women who have a history of incarceration may be further marginalized and have reduced access to reproductive health services. Incarceration is hypothesized to be associated with HIV acquisition [2], [13]. Pathways that lead from incarceration to increased HIV acquisition may be similar to those for pregnancy outcomes, and include marginalization and reduced access to healthcare. Although there has been much discussion of the impact of decriminalization of sex work on HIV incidence among FSW, the effects of decriminalization on sex worker health will likely extend beyond HIV.

More than half of women who reported a lifetime pregnancy also reported a history of unplanned pregnancy, which may indicate a large unmet need for family planning. Previous work has shown a high incidence of pregnancy among FSW [12], and a large proportion of FSW may have positive pregnancy intentions [14]. However, in our study, few (less than 2%) reported that they were currently trying to get pregnant as a reason for not using birth control. Although we did not specifically measure pregnancy or fertility desires in this study, this may be indicative that overall pregnancy intention is lower in this sample compared to previous work. The large percentage of women reporting unplanned pregnancy and termination of pregnancy indicates the urgent need for comprehensive reproductive healthcare for this population. For FSW populations, who frequently face large barriers to accessing healthcare, ensuring reproductive health services are accessible and providers are not stigmatizing will be essential to ensuring access to comprehensive care.

The results of this study must be considered in the context of several limitations. This survey relied on self-report for all measures. Self-reported history of pregnancy termination was likely underreported, and our estimated prevalence may therefore be an underestimate of the true abortion prevalence in this population. This study relied on cross-sectional data, so the temporality of the relationship between contraceptive use and reproductive health outcomes cannot be determined. Women enrolled in the ZEST study were not known to be living with HIV; as such, there may be important differences between women living with HIV and those with no known HIV infection that we are not able to detect in these analyses. Participants in our sample were women working in three Zambian transit towns of Livingstone, Chirundu, and Kapiri Mposhi, and may not be representative of the FSW population more broadly in Zambia or in sub-Saharan Africa. However, given similarities in the prevalence of contraceptive use [6], [11] and abortion [4], [15], [16], [17], [18], [19] in this study compared to other studies of FSW, these results may be generalizable to women in other similar transit settings in sub-Saharan Africa. However, despite these limitations, this study presents some of the first evidence of contraceptive use and reproductive health outcomes for FSW in Zambian transit towns.

In this study, we document high prevalence of unplanned pregnancy and pregnancy termination. Although approximately three-quarters of women were using some form of family planning, the prevalence of unwanted pregnancy underscores the need to expand FSW-friendly reproductive health services in transit towns in Zambia. The results of this study indicate that structural interventions, such as increasing condom availability in work places and decriminalizing sex work, may improve reproductive health outcomes for FSW in Zambia.
